# Supplementary material for: The causal relationship between the human gut microbiota and pyogenic arthritis: a Mendelian randomization study
Source: Front Cell Infect Microbiol. 2024 Nov 26;14:1452480. doi: 10.3389/fcimb.2024.1452480 (PMC11629706; doi:10.3389/fcimb.2024.1452480)
Supplement: Supplementary file 1 [file DataSheet1.zip › mendelian test/ebi-a-GCST90027564.csv_leave_one_out.pdf]

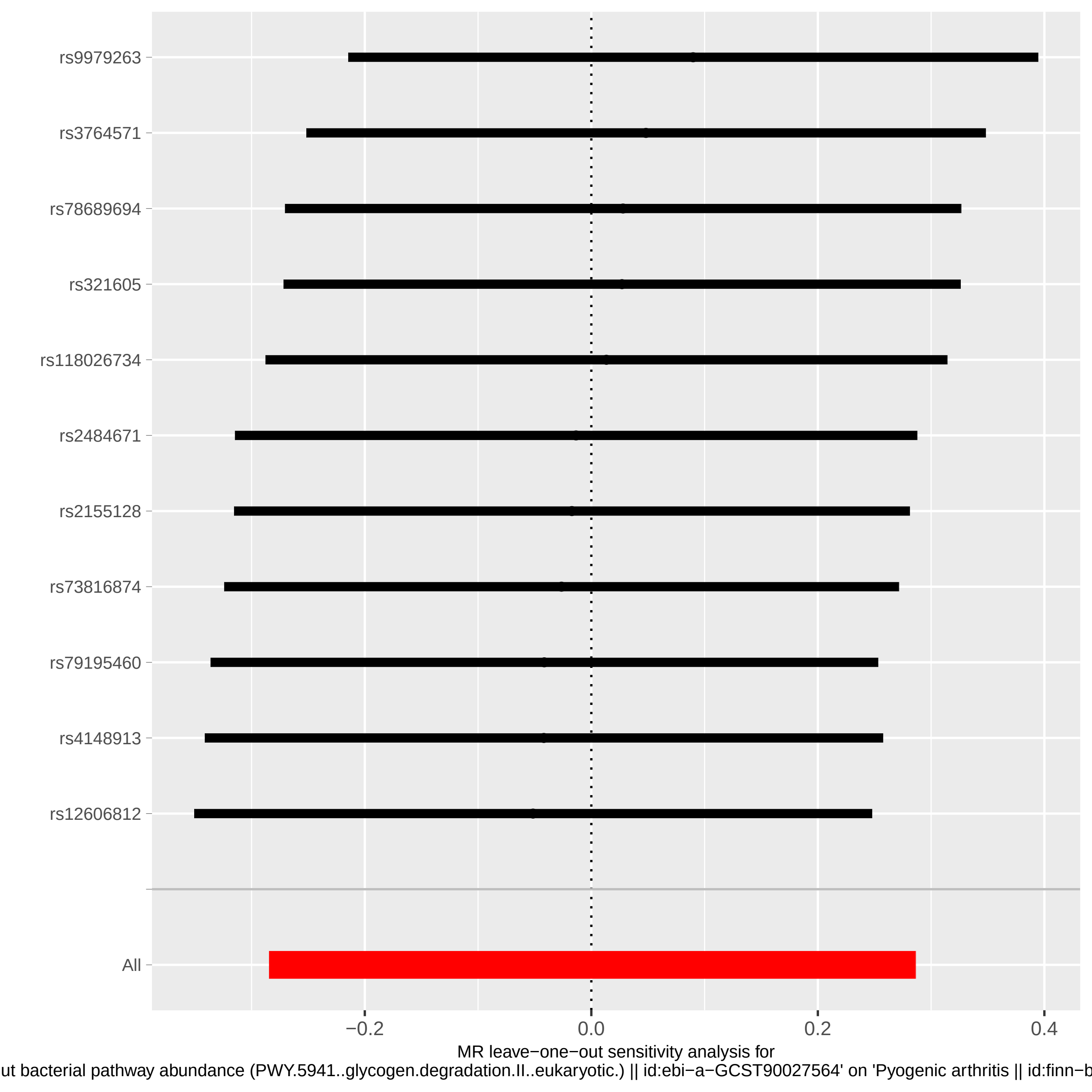
rs9979263
rs3764571
rs78689694
rs321605
rs118026734
rs2484671
rs2155128
rs73816874
rs79195460
rs4148913
rs12606812
All
−0.2
0.0
0.2
0.4
MR leave−one−out sensitivity analysis for
ut bacterial pathway abundance (PWY.5941..glycogen.degradation.II..eukaryotic.) || id:ebi−a−GCST90027564' on 'Pyogenic arthritis || id:finn−b
